# Homocysteine, Folic Acid, Cyanocobalamin, and Frailty in Older People: Findings From the “Invece. Ab” Study

**DOI:** 10.3389/fphys.2021.775803

**Published:** 2021-12-15

**Authors:** Antonio Guaita, Laura Brunelli, Annalisa Davin, Tino Emanuele Poloni, Roberta Vaccaro, Stella Gagliardi, Orietta Pansarasa, Cristina Cereda

**Affiliations:** ^1^Epidemiological and Neuropathological Laboratories, Golgi Cenci Foundation, Abbiategrasso, Italy; ^2^Department of Environmental Health Sciences, Istituto di Ricerche Farmacologiche Mario Negri IRCCS, Milan, Italy; ^3^Genomic and Post-Genomic Unit, IRCCS Mondino Foundation, Pavia, Italy; ^4^Department of Woman, Mother and Newborn, ASST Fatebenefratelli Sacco, “V. Buzzi” Children Hospital, Milan, Italy

**Keywords:** homocysteine, frailty, older people, cyanocobalamin, folic acid, longitudinal study

## Abstract

Frailty is an important age-related syndrome associated with several adverse health outcomes. Its biological basis is undefined. Raised plasma homocysteine (HOcy) is an established risk factor for cardiovascular disease, dementia, cognitive impairment, and mortality, but little is known about the possible role of plasma HOcy, cyanocobalamin (B12), and folate (FO levels in the development of frailty. Our first aim was to explore the possible association between frailty and plasma concentrations of HOcy, FO, and B12 in a cohort of community-dwelling older people. The second was to assess the influence of these metabolic factors on six-year incidence of frailty in the 875 individuals eligible for inclusion in this study (those with a full follow-up dataset). This research is based on data from three waves – 2012 (herein taken as baseline), 2014, and 2018 – of a longitudinal study (InveCe.Ab) in which non-frail men and women born between 1935 and 1939 underwent multidimensional assessments. Frailty was estimated using a deficit accumulation-based frailty index (FI). HOcy concentration was significantly positively correlated with FI at all timepoints, while B12 and FO levels were not. Plasma concentration of HOcy emerged as a predictor of six-year cumulative incidence of frailty, independent of age, sex, and education, while B12 and FO levels showed no relationship with frailty incidence. Individuals with plasma HOcy in the top quintile showed five months less frailty-free survival (HR 1.487; 95% CI: 1.063–2.078), regardless of age, sex, and education. These results demonstrate that higher HOcy is a risk factor for frailty onset in older adults.

## Introduction

Frailty is a complex geriatric syndrome associated, with biological vulnerability to stressors and decreased physiological reserve (Clegg et al., 2013). Among the possible factors influencing the onset of frailty, a biological action of homocysteine (HOcy) is plausible. (Ostrakhovitch and Tabibzadeh, 2019). Although frailty shares a common pathophysiological mechanism with aging, it is not necessarily related to the aging process and not every individual will experience frailty with aging (Topinková, 2008). Its etiology and pathogenesis are not completely understood, although various causes and complex pathways have been proposed. Several pathophysiological factors, including dysregulation of inflammatory processes, oxidative stress, mitochondrial dysfunction, and cellular senescence, underlie the frailty syndrome (Pansarasa et al., 2019), and it is also influenced by other factors, such as sociodemographic characteristics, psychological conditions, nutritional status, lack of physical activity, and comorbidities (Dent et al., 2016). Although it remains unclear what drives frailty, and little is known about the biological factors that contribute to the development of the syndrome (Ho et al., 2011), the latter can reasonably be thought to include raised plasma levels of HOcy. High plasma HOcy is associated with several conditions and circumstances, including older age, an unhealthy lifestyle, a poor diet, MTHFR 677C3T polymorphism, drug consumption, folate (FO) or cobalamin deficiency, diabetes, impaired renal function, and inborn errors of metabolism, such as homocystinuria (Refsum et al., 2004). HOcy concentration is a predictor of cardiovascular and all-cause mortality in the elderly (Bostom et al., 1999; Kark et al., 1999; Vollset et al., 2001); furthermore, with a few notable exceptions, most studies report hyperhomocysteinemia in people with dementia compared with healthy controls (Zhuo et al., 2011), and the hypothesis of a genetic influence (Roostaei et al., 2018), possibly acting more on executive functions than on memory (Polito et al., 2016), has also been advanced. HOcy may affect the aging process through endothelial dysfunction (Carluccio et al., 2007; Zhang et al., 2001), oxidative stress (Jacobsen, 2000), neurotoxicity (Ho et al., 2002), and DNA methylation status (Fuso et al., 2005). All these biologic pathways could lead to multisystem decline and a worse or accelerated aging process (Perez et al., 2007), in turn leading to frailty. The metabolism of HOcy is mainly dependent on its conversion to methionine by remethylation; this occurs *via* the main methionine synthase pathway, which requires FO and B12 coenzymes. Total plasma HOcy has been shown to be inversely related to the intake and plasma levels of FO and B vitamins, and HOcy level is considered to be an indirect biomarker of the metabolic action of these vitamins (Green, 2008).

Data on the influence of HOcy on frailty onset are still inconclusive; some authors found an association with prevalence on cross-sectional analysis (Wong et al., 2013), whereas others did not find this association (Matteini et al., 2008). Data on the role of B vitamins and FO in frailty are also inconsistent. Semba and colleagues (Semba et al., 2006), prospectively analyzing data from the Women’s Health and Aging Study I, concluded that there was no association between B vitamins and incident frailty after 3years of follow-up. Michelon and colleagues (Michelon et al., 2006) found a higher prevalence of vitamin B12 deficiency among frail as opposed to non-frail community-dwelling older women, but observed no apparent association between frailty and serum levels of B vitamins. Investigators using data from the InCHIANTI (Invecchiare in Chianti, aging in the Chianti area) study found that low FO intake was independently associated with frailty (Bartali et al., 2006a).

Although the above-reported results on the HOcy-frailty relationship highlight an association, none clarify the causal role or the extent of the frailty risk associated with altered HOcy, B12, and FO plasma levels in older people.

The present study was conducted to verify the proposed association of HOcy, B12, and FO plasma concentrations with frailty and to highlight the influence of these biomarkers on frailty incidence in a community-dwelling older population, studied longitudinally.

## Materials and Methods

### Study Design and Setting

The present research was conducted retrospectively within the framework of the InveCe.Ab (Invecchiamento Cerebrale in Abbiategrasso) study, a longitudinal multidimensional population study (ClinicalTrials.gov, NCT01345110). The design of InveCe.Ab is described in detail elsewhere (Guaita et al., 2013). Briefly, assessments were performed in 2010, 2012, 2014, and 2018 and involved individuals born between 1935 and 1939, recruited among the residents of Abbiategrasso, a small town near Milan. In each wave of the study, the participants underwent a multidimensional (social, medical, and neuropsychological) assessment, performed by trained interviewers, geriatricians, and neuropsychologists. In addition, they provided a blood sample for biological analyses. The evaluations and blood sampling took place in the research building of the Golgi Cenci Foundation, in the town center; in a few exceptional cases, the assessments were performed at home. The study procedures were in accordance with the Declaration of Helsinki, and the study protocol was reviewed and approved by the Ethics Committee of the University of Pavia on October 6^th^, 2009 (Committee report 3/2009). All the participants gave their written informed consent to the study.

### Participants

**Figure 1:**
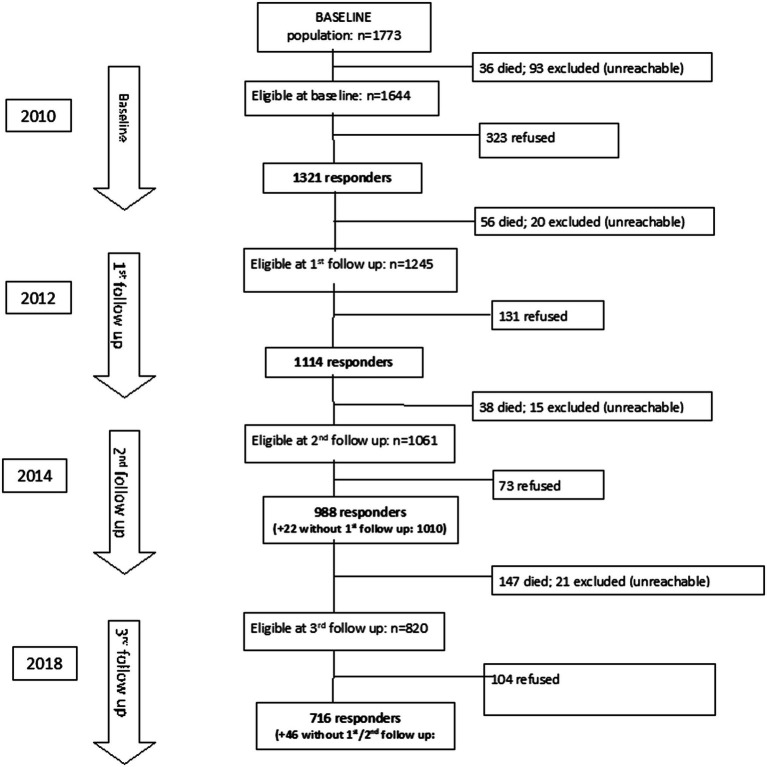
Flow chart of participants through the InveCe.Ab study 2010–2018 (4 waves).

The 2010, 2012, 2014, and 2018 waves of the InveCe.Ab included 1,321, 1,114, 1,010, and 762 subjects, respectively. Since plasma concentration of HOcy was available only from 2012, the present study was conducted using data from individuals participating in the second to the fourth assessments (2012–2018). No participant was under treatment for renal impairment. All participants with full datasets were included in cross-sectional analyses, while only those who had completed at least two assessments that included calculation of an index of frailty and blood tests (see Figure 1 for the study flow chart) were eligible for inclusion in longitudinal analyses. The InveCe.Ab study participants were aged between 73 and 77 at the first assessment (2012) and between 78 and 83 at the last (2018).

### Variables

#### Frailty

Frailty was estimated using a frailty index (FI), calculated on the basis of accumulation of deficits, a method widely used in older adults (Mitnitski et al., 2001). In our protocol, the FI was based on a list of 32 health variables (Table 1). Each of these 32 items was assigned a score of 0 or 1, where 0=absence and 1=presence of the deficit. Only one item (body mass index) was coded in three classes. Each participant’s scores were then combined in a single index by dividing their number of deficits (ranging from 0 to 32) by the total number considered (32). A final index of 1 corresponded to maximum frailty. On the basis of the FI values obtained, the participants were categorized into three groups: Fit (FI≤0.08), PreFrail (FI between 0.08 and 0.25), and Frail (FI≥0.25), as reported in the literature (Song et al., 2010). In some of our analyses, FI classes were dichotomized as Frail versus Non-Frail (PreFrail+Fit).

**Table 1:** Frailty index (FI) items.

Comorbidities		Body mass index, kg/m2	
Hypertension	1	18.5–24.9	0
Congestive heart failure	1	25.0–29.9	0.5
Coronary artery disease	1	<18.5 or>30.0	1
Cardiac arrhythmia	1	**Functional items**	
Hyperlipidemia	1	Problems with cooking	1
Stroke	1	Problems with eating	1
Arthritis	1	Problems with dressing	1
Asthma	1	Toileting problems	1
Cancer	1	Housekeeping problems	1
Chronic kidney disease	1	Problems with bathing	1
Chronic obstructive pulmonary disease	1	Impaired mobility	1
Depression	1	Problems using transportation	1
Diabetes mellitus	1	Difficulty getting out of bed	1
Osteoporosis	1	Use of medications	1
Thyroid disease	1	Polypharmacy (> 5)	1
Liver disease	1	Walking dependency	1
Substance abuse in the last week	1	Urinary incontinence	1
Falls	1	TOTAL	0–32

*The FI is the sum of the individual’s scores divided by the total number of items considered*.

#### Homocysteine, B12, Folate

Clinical biochemistry assays on EDTA plasma samples were performed at each assessment wave, and they included a list of biomarkers relevant to the purposes of the InveCe.Ab study. All blood samples were taken in the morning from fasting subjects in a sitting position, a condition that may induce an up to 10% increase in HOcy concentration (Rasmussen and Moller, 2000). Among these biomarkers, the present study considered data on plasma levels of HOcy, B12, and FO, which had been analyzed by chemiluminescent microparticle immunoassay on an Architect 2000 analyzer (Abbott, Abbott Park, IL, United States) within 3h of blood collection. We set 19.4μmol/l as the cutoff plasma concentration of HOcy, a value identifying the 75^th^ percentile in the eligible subjects (1,077 individuals) and, approximately, the top quintile in those included in the longitudinal study (875). After defining the cutoff for the 80^th^ percentile (new variable: HOcy80), the HOcy values were coded in two classes: lower (<19.4μmol/l) and higher (≥19.4μmol/l). A further variable, where the cutoff value of 23.3μmol/l corresponded to the 90^th^ percentile (HOcy90), was also introduced.

#### Other Variables

The sociodemographic variables considered in this study were sex, age (at the baseline medical examination), and education (years of schooling completed).

### Statistical Methods

Descriptive statistics were used to analyze the features of the population, reporting mean values with standard deviation for normally distributed variables, medians with interquartile range for non-normally distributed variables, and percentages for nominal variables. Data from the first assessment (2012), taken as baseline for the present study, were reported by comparing the included and the excluded subjects; instead, descriptive data comparing Frail and Non-frail individuals were reported for the next two waves (2014 and 2018). In both cases, differences were analyzed using Student’s t test to compare mean values and the Mann–Whitney U (non-parametric rank) test to compare non-normally distributed variables.

#### Cross-Sectional Analyses

Relationships between FI as a continuous variable and HOcy, B12, and FO were tested with Spearman’s rho for each assessment wave.

#### Longitudinal Analyses

Logistic regression analysis, with age, gender, and education as covariates, was performed to test the influence of baseline plasma HOcy concentration and HOcy class on the cumulative incidence of frailty. Due to their collinearity with HOcy, the influence of B12 and FO on FI cumulative incidence was analyzed with logistic regression, in a single separate model. To highlight the influence of the two plasma HOcy concentration classes on the time to onset of frailty, Kaplan-Meyer survival curves were applied to explore frailty-free survival time. This aspect was also analyzed with Cox hazard regression models, introducing age, sex, and education as covariates. Statistical tests were computed using SPSS version 20.0 (SPSS, Chicago, IL, United States), setting the significance threshold at *p*<0.05.

## Results

### Descriptive Features of the Study Participants

Descriptive analyses were performed on the 2012 wave data (taken as baseline). The participants examined for eligibility numbered 1,114; in 14 of these, the FI could not be calculated due to missing data, and among these, 38 had no blood sample; so, 1,062 individuals were examined at baseline for reporting descriptive data. Table 2 shows the characteristics of the included versus the excluded individuals at baseline. The enrolled participants were younger and showed lower HOcy and higher FO plasma concentrations, while B12 plasma levels did not differ between the two groups (Table 2). For longitudinal analysis, a further 109 were excluded because they were already frail, 75 because they did not meet the criterion of two evaluations, and 3 because no blood sample data were available. Therefore, 875 subjects were included in the longitudinal examination. None of them had renal insufficiency. Of these participants, 858 still met our inclusion criteria at the 2014 follow-up and 670 at the 2018 one. The mean observation period was 5.85years. The enrolled subjects with HOcy values over 19.4 (HOcy80) and over 23.3 (HOcy90) μmol/l numbered 196 and 89, respectively.

**Table 2:** Features of the baseline eligible population comparing included and excluded subjects.

	Total (n=1,062) Females 52.6%	Included (n=875) Females 53%	Not included (n=187) Females 52.2%	*p*
Age, mean in years (SD)	74.34 (1.360)	74.30 (1.349)	74.56 (1.391)	0.018
Education, mean in years (SD)	6.75 (3.363)	6.79 (3.364)	6.58 (3.364)	0.425
HOcy, median (IQR) μmol/l	15.70 (13.00–19.40)	15.50 (13.00–18.70)	16.60 (13.50–21.00)	0.001
B12, median (IQR) μmol/l	364.00 (280.00–474.00)	365.00 (282.00–462.00)	363.00 (260.00–503.00)	0.983
FO, median (IQR) μmol/l	5.10 (3.90–7.300)	5.20 (3.97–7.60)	4.70 (3.20–6.80)	0.002

*The mean values with standard deviation (SD) were compared using Student’s t test, while the median values with interquartile range (IQR) were compared using non-parametric rank analyses (the Mann–Whitney U test). Gender distribution did not differ significantly between the two groups. FI is not reported as the absence of fragility was the baseline selection criterion in this population*.

**Figure 2:**
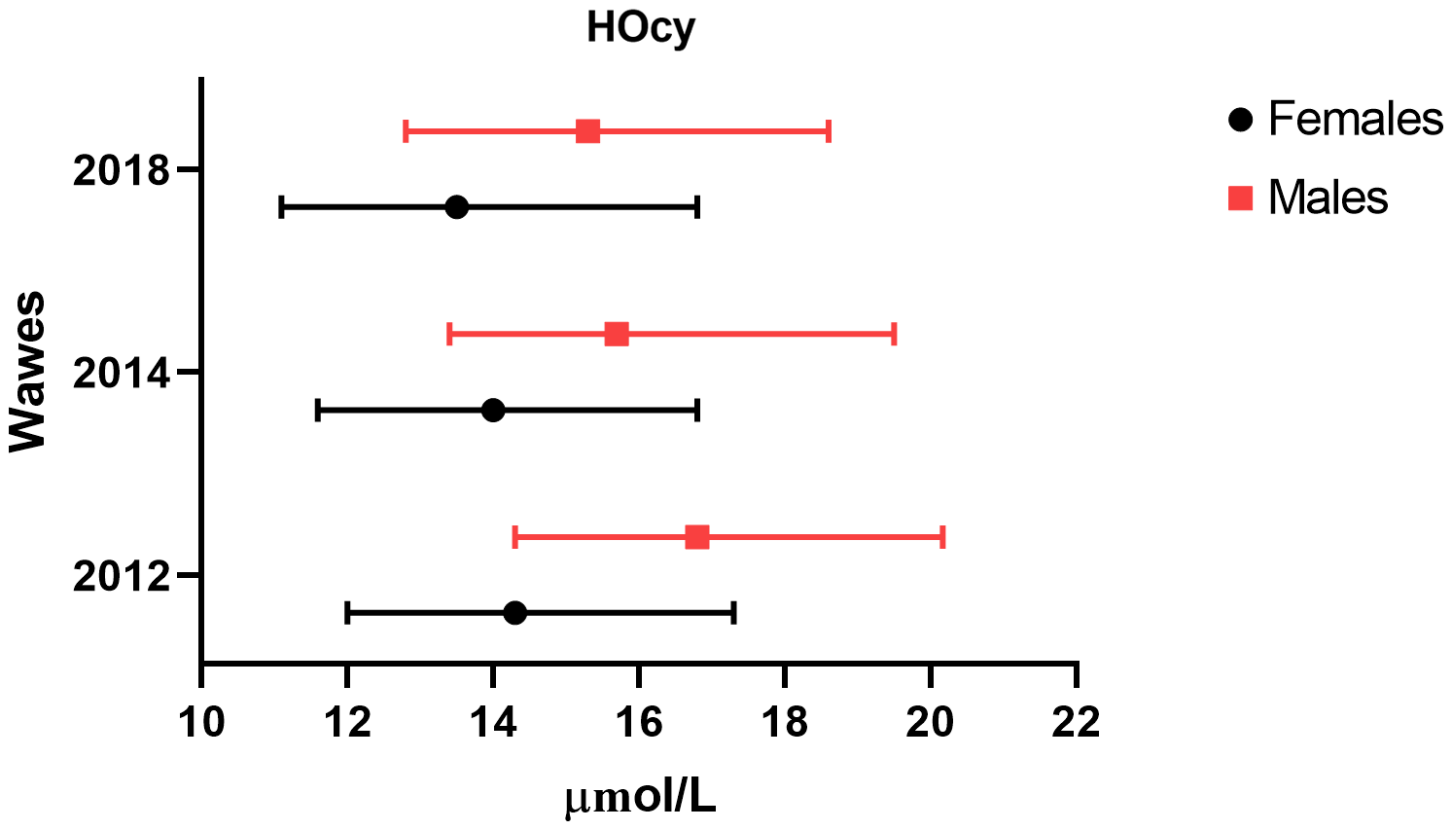
Sex difference in plasma HOcy concentration (μmol/l). Median and interquartile ranges of HOcy reported by sex. All differences were statistically significant (*p*<0.01; Mann–Whitney U).

Descriptive data of the 2012, 2014, and 2018 participants were analyzed, comparing features of the Frail and the Non-frail subgroups. Frail individuals accounted for 7.3% of the 2014 and 22.2% of the 2018 participants. (Table 3). In the 2014 wave, age and HOcy concentration were significantly higher in the Frail subjects, while there were no differences in FO or B12 plasma concentrations. In the 2018 wave, no significant differences in these biomarker levels were found between the Frail and the Non-frail groups (Table 3). There was no significant difference in the sex distribution at any of the timepoints, but HOcy concentration was significantly higher in the male participants at each of them in consideration of these results, we introduced sex as a covariate in all the longitudinal analyses (Figure 2).

**Table 3:** Characteristics of the 2014 and 2018 subjects, recruited for longitudinal analyses comparing the Frail and the Non-frail groups.

	2014 assessment				2018 assessment			
	Total (n=858)	Frail (n=63)	Non-frail (n=795)	p	Total (n=670)	Frail (n=149)	Non-frail (n=521)	p
Age, mean in years (SD)	76.22 (1.402)	76.60 (1.351)	76.19 (1.403)	0.026	80.77 (1.3647)	80.82 (1.397)	80.75 (1.356)	0.609
Education, mean in years (SD)	6.78 (3.363)	6.79 (3.385)	6.78 (3.363)	0.980	6.78 (3.314)	6.69 (3.312)	6.81 (3.318)	0.709
HOcy. median (IQR) μmol/l	14.90 (12.50–18.40)	15.85 (13.70–19.65)	14.800 (12.50–18.20)	0.030	14.30 (11.80–17.70)	14.20 (12.50–18.70)	14.30 (11.75–17.50)	0.377
B12, median (IQR) nmol/l	322.00 (250.00–418.00)	285.00 (235.25–439.75)	327.00 (252.00–417.00)	0.199	293.00 (229.00–385.00)	291.00 (220.50–376.75)	293.00 (232.00–385.75)	0.381
FO, median (IQR) μmol/l	5.15 (3.80–7.33)	4.75 (3.73–7.78)	5.20 (3.90–7.20)	0.738	5.06 (3.87–6.98)	5.06 (3.70–7.24)	5.09 (3.90–7.01)	0.695

*The mean values with standard deviation (SD) were compared using Student’s t test, while the median values with interquartile range (IQR) were compared using non-parametric rank analyses (the Mann–Whitney U test)*

### Cross-Sectional Analyses

The participants with full data sets for the 2012, 2014, and 2018 assessment waves, who were therefore included in the cross-sectional analyses, numbered 1,062, 990, and 786, respectively. Using Spearman’s rho rank correlation coefficient, the relationships between FI as a continuous variable and HOcy, HOcy80, HOcy90, B12, and FO were examined taking into account the 2012, 2014, and 2018 assessment wave data from all the participants. HOcy was significantly positively correlated with FI at all the waves of the study (Table 4). B12 and FO did not correlate with FI at any wave, whereas they correlated strongly with HOcy (data not reported in the Table).

**Table 4:** Rank correlation coefficient between plasma HOcy concentration (μmol/l) and FI at the three waves.

Wave	N of participants included	Spearman’s rho	p
2012	1,062	0.075	0.015
2014	990	0.079	0.013
2018	786	0.081	0.030

*Rank correlation results (Spearman’s Rho) were reported for each wave with the number of subjects (N) involved and the statistical significance (p)*.

### Longitudinal Analyses

The relationships between FI as a continuous variable and HOcy, HOcy80, HOcy90, B12, and FO in the enrolled subjects were then examined longitudinally.

#### Hocy, HOcy80, and HOcy90 as Predictors of Cumulative Incidence of Frailty: Logistic Regression Analysis

The cumulative six-year incidence of frailty was 184/875 (21%). A logistic regression model with cumulative incidence of frailty as a dependent variable, HOcy as a predictor, and sex, age, and education introduced as covariates, showed a positive relationship (OR:1.028; 95%CI: 1000–1.052; *p*=0.048). On adopting HOcy80 as a predictor of cumulative incidence of frailty, only a positive trend was shown (OR: 1.412; 95% CI: 0.963–2.069; *p*=0.077). On the contrary, the regression model with HOcy90 as a predictor showed a statistically significant influence (OR: 1.878; 95%CI: 1.153–3.059; *p*=0.011). Applying the same model, B12 and FO, which cross-sectional analyses had not shown to be related to FI, continued to show no relationship with frailty incidence on binary logistic regression analysis (B12: OR 1.000; 95%CI: 0.999–1,001; *p*=0.611. FO: OR 1.005; 95%CI: 0.969–1,043; *p*=0.774).

#### HOcy80 And HOcy90: Influence on Frailty-Free Survival Time

At baseline (2012 wave), 196 subjects were in the higher HOcy80 class (top quintile) and 679 in the lower one. Kaplan–Meier survival analysis was applied to compare the frailty-free months from baseline for these two classes. Table 5 reports the mean frailty-free time (in months) for each of them. The individuals with the higher HOcy80 values had five fewer months free from frailty. The Mantel-Cox (Log Rank) comparison was statistically significant (p=0.048).

**Table 5:** Frailty-free survival time according to HOcy80 plasma concentration.

Homocysteine (HOcy80) plasma concentration class	N	Frailty incidence (n of events)	Frailty-free months (mean)	95% Confidence Interval	
				Lower Bound	Upper Bound
lower (<19.4μmol/l)	679	134	74.8	73.5	76.1
higher (≥19.4μmol/l)	196	50	69.8	66.9	72.8

*Frailty incidence (n of events) and the mean number of frailty-free months were reported for the two HOcy80 classes. The Mantel-Cox analysis evidenced a statistically significant difference (p=0.048)*.

**Figure 3:**
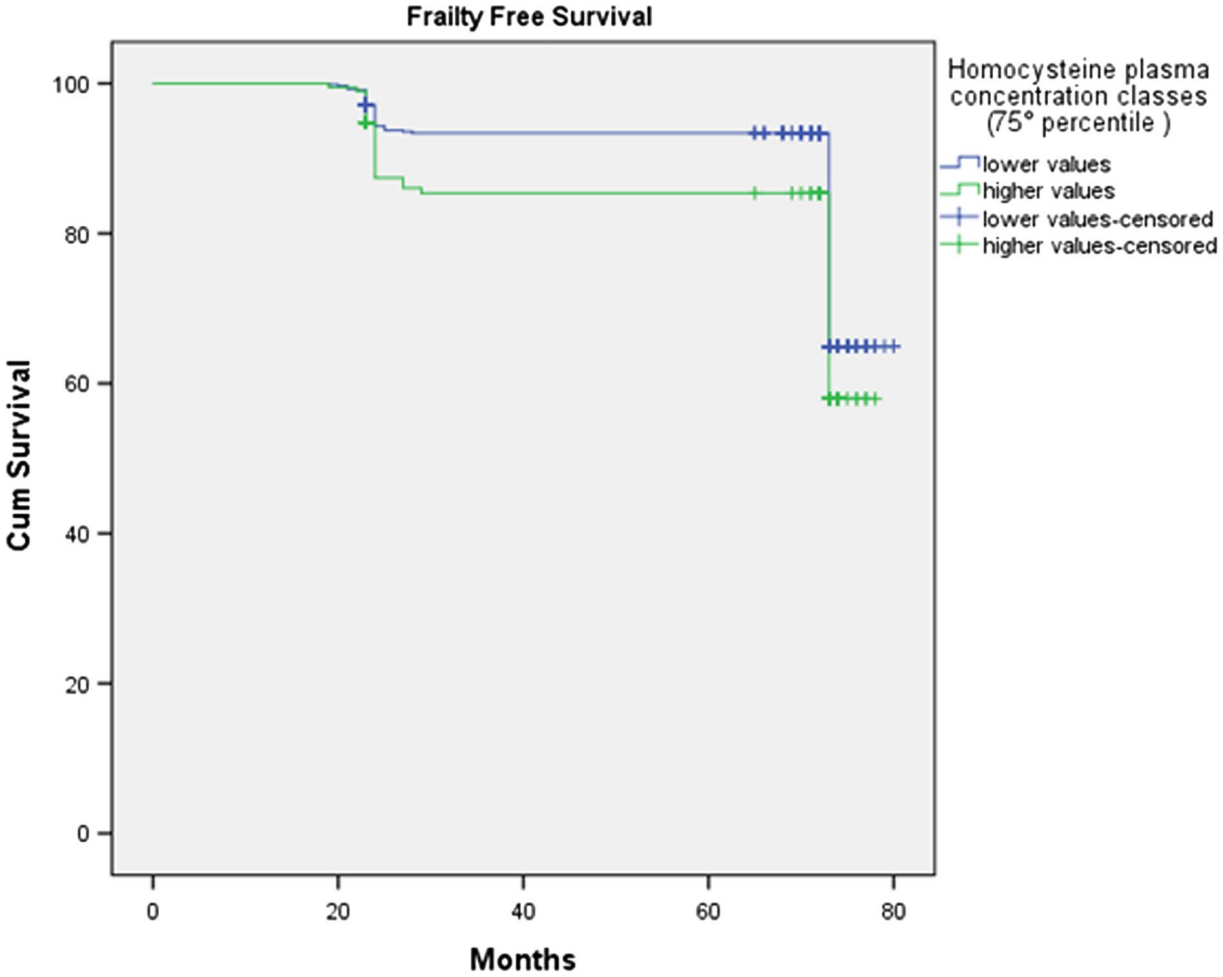
The survival plot shows that subjects with lower HOcy plasma concentration values display longer frailty-free survival (upper line).

The survival plot is shown in Figure 3. In the survival plot, two separate lines can be seen to track frailty-free survival in the two HOcy80 classes, with the subjects with lower HOcy values constantly represented by the upper line, which at no point crosses the lower one.

The results of the Cox Proportional Hazard model, in which age, sex, and education were again introduced as covariates, were in the same direction. We preliminarily tested the assumptions and checked that each variable fulfilled the proportional assumption. The results are shown in Table 6. HOcy80 significantly influenced frailty-free time (HR 1.487; 95%CI 1.063–2.078; *p*=0.020). The introduced covariates were all insignificant and did not contribute to the model.

**Table 6:** Cox proportional hazard model.

	B	p	HR	95% CI of HR	
				Lower	Upper
HOcy80	0.397	0.020	1.487	1.063	2.078
Age	−0.083	0.126	0.920	0.827	1.024
Sex	0.167	0.275	1.181	0.876	1.593
Education	−0.001	0.963	0.999	0.956	1.044

*Dependent variable: Frail/Non-frail. For each variable introduced in the model, the beta values (B) and the significance (p) of the Cox regression were reported, along with the hazard ratio (HR) with 95% confidence interval (CI)*.

With regard to the HOcy90 plasma concentration classes (cutoff: 23.3μmol/l), 89 subjects were classified in the higher class and 786 in the lower one. Kaplan–Meier survival analysis was applied to compare the frailty-free months from baseline between the two groups. The individuals with a higher HOcy90 plasma concentration had five fewer frailty-free months. The Mantel-Cox (Log Rank) comparison was statistically significant (p=0.02; Table 7). The HOcy90 survival plot lines (not reported) were similar to the HOcy80 ones, while the Cox regression results showed a higher HR (1.641; 95%CI: 1.090–2.469; *p*=0.018).

**Table 7:** Frailty-free survival time for the two HOcy90 plasma concentration classes.

Homocysteine plasma concentration class	N	Frailty incidence (n of events)	Frailty-free months (mean)	95% Confidence Interval	
				Lower Bound	Upper Bound
lower (<23.3μmol/l)	786	156	74.2	73.0	75.5
higher (≥23.3μmol/l)	89	28	69.2	65.2	73.1

*Frailty incidence (n of events) and the mean numbers of frailty-free months were reported for the two HOcy90 classes. The Mantel-Cox analysis showed a statistically significant difference (p=0.02)*.

## Discussion

The main goal of this research was to analyze the influence of plasma HOcy concentration on the development of frailty in older people. The main results are summarized in the following list.

- The proportion of frail subjects increased from 7.3% in the 2014 wave to 22.2% in the 2018 wave of the study.

- The median concentration of HOcy increased with age only in males.

- HOcy showed a significant positive correlation with FI at all three waves of the study, while B12 and FO did not.

- HOcy emerged as a predictor of six-year cumulative incidence of frailty, independent of age, sex, and education, although HOcy80 showed only a positive trend in this sense. On the contrary HOcy90 significantly influenced the cumulative incidence of frailty.

- B12 and FO showed no relationship with the cumulative incidence of frailty.

- Individuals with plasma HOcy concentrations in the top quintile (HOcy80) had five fewer frailty-free months (HR 1.487; 95% CI: 1.063–2.078); this finding was independent of age, sex, and education. A comparable frailty-free survival time, but with a higher HR (1.641; 95% CI:1.090–2.469), was found for plasma HOcy concentrations in the top decile (HOcy90). These results support the hypothesis that HOcy is a risk factor for the occurrence of frailty.

Comparing our results with those already reported in the literature is problematic, for reasons that help us to highlight the strengths and limitations of the present study.

First of all, prior to our study, the relationship between HOcy and frailty in older people had been analyzed longitudinally in just two studies (Wong et al., 2013; Swart et al., 2013), with inconclusive results; all other published studies have been cross-sectional.

Second, we assessed frailty using an index based, in accordance with the indications of Searle and collaborators (Searle et al., 2008), on the indvidual’s accumulation of deficits. The FI is a commonly used method, but differs from other widely used tools (Rockwood et al., 2007), such as Linda Fried’s “frailty phenotype” (Fried et al., 2001), which is defined by five items: four performance ratings and a weight loss item. The fact that there is no way of knowing whether plasma HOcy is a risk factor also for frailty measured with other tools could explain why the few cross-sectional studies dealing with the relationship between HOcy and frailty in older people have led to discrepant results and conclusions (even though most found a positive association). The FRAIL index adopted by the Australian researchers conducting the Health in Men Study identified an association between prevalence of frailty and HOcy concentrations over 15μmol/l (Wong et al., 2013; van Kan et al., 2008). On the contrary, a cross-sectional study in older women, in which frailty was assessed using a “frailty phenotype index” and the level of “normal” HOcy was set at 13.9μmol/l, no relationship was found between plasma HOcy concentration and frailty (Matteini et al., 2008). Similarly, a Taiwanese study failed to find an association between HOcy and frailty, assessed with the five-item “frailty phenotype” (Hwang et al., 2015).

In the Toledo study, which involved both sexes, a cross-sectional analysis was performed to test the association between frailty, defined according to the “frailty phenotype” and HOcy. HOcy was independently associated with frailty [odds ratio (OR)=1.06; 95%CI: 1.01–1.12] (Álvarez-Sánchez et al., 2021). The Rugao Longevity and Ageing Study, involving 1,480 individuals of both sexes, found HOcy to be significantly associated with frailty, defined using Fried’s phenotype criteria, with an OR of 2.27 (95%CI 1.36–3.78) for high HOcy after adjusting for multiple confounding factors. In the conclusion of that study, in which genetic variants were also considered, the authors define HOcy as a marker and not a possible causal factor of frailty (Ma et al., 2020).

In the Longitudinal Aging Study Amsterdam, HOcy was studied in relation to grip strength and functional limitations, which can be considered proxies of frailty. HOcy values in the fourth quartile (17.75μmol/l for men and 15.72μmol/l for women), compared with the first, were significantly associated with functional limitations in both sexes. In the longitudinal analysis, only women showed a relationship between HOcy and onset of functional limitations (Swart et al., 2013).

In the longitudinal analysis of data from the Women’s Health and Aging Study I, women showed a significant relationship between serum vitamin B6 and B12 levels and incident disability, which may be considered a functional correlate of extreme frailty. The authors also reported association between serum HOcy levels and incident disability, although no data were provided (Bartali et al., 2006b). Wong and collaborators in the already mentioned Australian study, applying longitudinal analysis, found no influence of high HOcy on frailty onset in the male population analyzed (Wong et al., 2013). None of the previous studies assessing the influence of HOcy on frailty in older adults adopted the “Frailty Index.”

A third point concerns the different HOcy concentrations used as cut-off values to obtain normal and “high” classes. In the aforementioned studies, the cutoff levels, when reported, were different from and in general lower than the 19.4 μmol/l and 23.3 μmol/l used as cutoffs in our study. It may be incorrect to take clinical cutoffs referring to general populations of any age and apply them to a specific population of older people, as mean HOcy has been shown to increase with aging (Moustapha and Robinson, 1998; Azzini et al., 2020). This age-associated increase may be independent of specific pathologies and linked to declining renal function, nutritional deficiencies, deregulation of the methionine cycle and deficiencies of HOcy remethylation and trans-sulfuration, all cofactors contributing to elevation of HOcy with advancing age (Ostrakhovitch and Tabibzadeh, 2019). Xu and collaborators found that HOcy concentration increased markedly after 50years of age, reporting means (SD) of 13.90 (5.48) μmol/l in 50-year-old males, 16.32 (6.43) μmol/l in 60- to 80-year-olds, and 18.75 (6.16) μmol/l in the >80years age group (Xu et al., 2020). The results of our study, which showed a mean HOcy level of 16.53 (SD: 5.71) μmol/l in people aged between 79 and 83years, are similar to those reported by Xu and collaborators (Xu et al., 2020), as well as those found in over-65s by McMahon and collaborators (McMahon et al., 2006). In the opinion of European experts, the possible role of age and comorbidities in increasing plasma HOcy levels should be taken into account whenever, both in clinical and in research settings, it is necessary to evaluate possible detrimental effects of HOcy or apply threshold levels of its plasma concentration (Refsum et al., 2004). In our study, the overall median value of HOcy did not increase significantly over the three waves of the study, and the difference, in HOcy values, between the Frail and the Non-frail groups narrowed over time. This can be explained by the “plateau” that was seen in the oldest old (as also observed by others in this age group), which may be a consequence of higher mortality in individuals with higher plasma HOcy concentrations (Wong et al., 2013; Mendonça et al., 2018). In the supplementary data we report the Kaplan–Meier survival curve for baseline HOcy80. Participants in the higher HOcy class showed a shorter survival than those with lower HOcy values (69.59 versus 73.56), a non-statistically significant difference (Log Rank, Mantel-Cox=1,701; *p*=0.192; Supplementary Table S1, Supplementary Figure S1).

Had we applied some of the other previously reported cutoff levels, such as 15 or even 13μg/l, most of our participants would have been in the “higher” plasma HOcy concentration class. The criteria adopted in the present study –we considered the value of the top quintile, or even the top decile, of the population eligible for longitudinal analysis, are similar to those applied in previous studies on HOcy as a cardiovascular risk factor in older people (Cheng et al., 1997; Bots et al., 1999).

The fourth key point worth discussing is the possible role of gender. Our study population included both sexes, with a slight prevalence of women, whereas some previous studies have enrolled only men or women. We found lower HOcy values in the women, as expected (Jacques et al., 1999; Refsum et al., 2004). Indeed, women have been proven to have higher rates of HOcy remethylation (Fukagawa et al., 2000). This difference was found to be slightly less marked with advancing age, with median differences of 2.5, 1.7, and 1.8μg/l observed at the 2012, 2014, and 2018 assessments, respectively. Others have found a reduced sex difference with increasing age (Jacques et al., 1999). The gender difference in HOcy values remained unchanged across the waves of the study; sex was introduced as a covariate in all the longitudinal analyses, although none of them showed it to exert an independent influence on our key outcome.

None of the previous studies dealing with HOcy and frailty has reported data on the influence of plasma HOcy concentration on frailty-free survival time. This is an important issue, not only as a new angle of research, but also for its implications for the lives of older people themselves: for everyone, becoming frail sooner in old age is certainly quite different from becoming frail later. Our Kaplan–Meier survival analysis showed, in relation to plasma HOcy status, a mean survival difference of five months in a period of less than six years. It should be emphasized that our study concerned over-80s, in whom life expectancy is short and five months can be considered a noteworthy period of time. The present study found no statistical association between B12 or FO and frailty; unsurprisingly, therefore, there was also no influence on six-year incidence of frailty, although plasma levels of these vitamins correlated strongly with HOcy. These data are similar to those of another prospective study, which instead analyzed three-year incidence of frailty in a female population (Semba et al., 2006). These authors, too, concluded that frailty showed no relationship with these vitamins. In the same vein, cross-sectional studies have also been unable to provide conclusive data on a role for B12 and FO in frailty. Other studies analyzed possible associations of vitamins with frailty only indirectly, by examining differences in plasma concentrations between frail and non-frail subjects (Michelon et al., 2006), or the independent role of dietary FO intake (Bartali et al., 2006a). On the other hand, the relationship between FO and B12 concentrations and absolutely relevant outcomes like mortality still remains to be demonstrated (Dangour et al., 2008; Bates et al., 2010), while beneficial effects of lowering HOcy through B12 and FO administration do not include an effect on survival (Clarke et al., 2010; Debreceni and Debreceni, 2012) or cognitive performances (McMahon et al., 2006). Although, on the one hand, our data are consistent with the findings of these studies, indicating a marginal role of B12 and FO as possible protective factors against frailty; on the other, they suggest that high HOcy concentrations are a hallmark as well as a risk factor for frailty, related to complex metabolic dysfunctions, inherent in the very concept of frailty as defined by the FI. Increased HOcy favors the onset of frailty, but frailty may promote increased plasma HOcy concentrations. Considering this, it is not surprising that HOcy lowering through vitamin intake is not sufficient to avoid the negative outcomes associated with high plasma HOcy concentrations.

The findings of this study should be interpreted taking into account some limitations. They refer to individuals who fall within a narrow range of advanced age and may therefore not necessarily be extended to people in younger ranges of old age. Similarly, all the participants were inhabitants of a small area in Italy, and all were Caucasian, and it must therefore be considered that individuals from different areas or ethnic groups might show different results. Furthermore, we were only able to consider a small number of covariates. On the other hand, this study also has several strengths. These are the first longitudinal data in a population of this age, moreover studied for six years and with two follow-ups from baseline; the population also had good number of participants of both sexes. This is the first study to adopt a FI, based on accumulation of deficits, to measure frailty in relation to HOcy, FO, and B12 concentrations. This, unlike other methods, allowed a continuous variable to be built and analyzed. Finally, these are the first data on the influence of plasma HOcy concentration on frailty-free survival time, which is a key outcome in this age group.

## Conclusion

Higher plasma HOcy concentrations, being a factor that increases the likelihood of becoming frail and becoming frail sooner, showed a clear association with frailty. Instead, the role of B12 and FO plasma concentrations in this setting was found to be marginal. These data need to be confirmed in more extensive research designs, taking into account more contextual elements, such as diet and lifestyle, and considering other subgroups, such as different ethnic groups, and “young” old people.

## Data Availability Statement

The raw data supporting the conclusions of this article will be made available by the authors, without undue reservation.

## Ethics Statement

The studies involving human participants were reviewed and approved by Ethics Committee of the University of Pavia on October 6th, 2009 (Committee report 3/2009). The patients/participants provided their written informed consent to participate in this study.

## Author Contributions

AG, LB, and AD conceived and designed the study. Clinical samples and data were provided by TP, RV, and AG. Statistical analyses were done by AG and LB who interpreted the results and wrote the draft. TP, RV, SG, OP, and CC contributed in interpreting the results and supervised the manuscript. Acquisition of funding was done by CC and AG. All authors have read and agreed to this published version of the manuscript.

## Conflict of Interest

The authors declare that the research was conducted in the absence of any commercial or financial relationships that could be construed as a potential conflict of interest.

## Publisher’s Note

All claims expressed in this article are solely those of the authors and do not necessarily represent those of their affiliated organizations, or those of the publisher, the editors and the reviewers. Any product that may be evaluated in this article, or claim that may be made by its manufacturer, is not guaranteed or endorsed by the publisher.

## Supplementary Material

The Supplementary Material for this article can be found online at:

https://www.frontiersin.org/articless/10.3389/fphys.2021.775803/full#supplementary-material
