# Supplementary material for: Homocysteine, Folic Acid, Cyanocobalamin, and Frailty in Older People: Findings From the “Invece. Ab” Study
Source: Front Physiol. 2021 Dec 15;12:775803. doi: 10.3389/fphys.2021.775803 (PMC8717775; doi:10.3389/fphys.2021.775803)
Supplement: Supplementary file 1 [file Table_1.docx]

**Supplementary material**

Table S1 Survival time according to HOcy80 plasma concentration.

| Homocysteine (HOcy80) plasma concentration class | N | mortality  (n of events) | Survival months (mean) | 95% Confidence Interval | |
| --- | --- | --- | --- | --- | --- |
|  |  |  |  | Lower Bound | Upper Bound |
| lower (<19.4 μmol/l) | 679 | 119 | 73.56 | 72.04 | 75.09 |
| higher (>19.4 μmol/l) | 196 | 41 | 69.59 | 66.35 | 72.84 |

*Death incidence (n of events) and the mean number of survival months were reported for the two HOcy80 classes.* *The difference was not statistically significant ( Log Rank ,Mantel-Cox=1,701; p= 0.192).*

Figure S1 Kaplan-Meier survival plot, comparing survival time of individuals with higher and lower homocysteine concentration class.

*The survival plot shows that subjects with lower HOcy plasma concentration values display longer frailty-free survival upper line, though the difference was not statistically significant*
