# Supplementary figures and images for: Homocysteine, Folic Acid, Cyanocobalamin, and Frailty in Older People: Findings From the “Invece. Ab” Study
Source: Front Physiol. 2021 Dec 15;12:775803. doi: 10.3389/fphys.2021.775803 (PMC8717775; doi:10.3389/fphys.2021.775803)

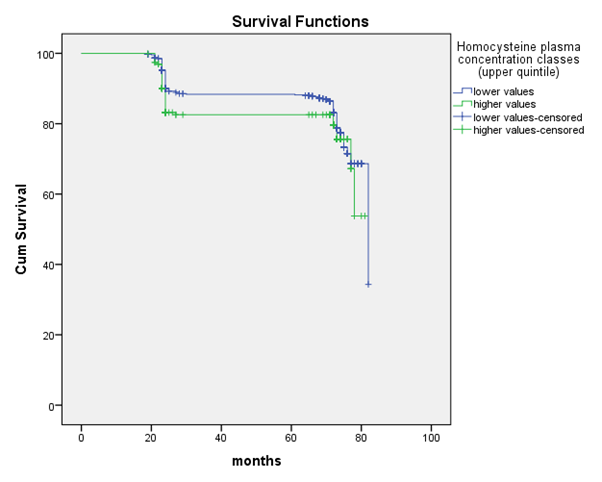

Supplement: Supplementary file 2 [file image_1.tif]
